# Supplementary material for: Deconstructing sarcomeric structure–function relations in titin-BioID knock-in mice
Source: Nat Commun. 2020 Jun 19;11:3133. doi: 10.1038/s41467-020-16929-8 (PMC7305127; doi:10.1038/s41467-020-16929-8)
Supplement: Supplementary file 1 — Supplementary Information [file 41467_2020_16929_MOESM1_ESM.pdf]

Supplement

Deconstructing sarcomeric structure–function relations in titin-BioID knock-in mice.

Franziska Rudolph[1], Claudia Fink[1], Judith Hüttemeister[1], Marieluise Kirchner[2], Michael H. Radke[1,6], Jacobo Lopez Carballo[1], Eva Wagner[3,4,5], Tobias Kohl[3,4,5], Stephan E. Lehnart[3,4,5], Philipp Mertins[2,7], & Michael Gotthardt[1,6,8*]

[1]*Neuromuscular and Cardiovascular Cell Biology, Max Delbrück Center for Molecular Medicine in the Helmholtz Association, Robert Rössle Str. 10, 13125 Berlin, Germany*

[2]*Proteomics Platform, Max Delbrück Center for Molecular Medicine in the Helmholtz Association, Robert Rössle Str. 10, 13125 Berlin, Germany*

[3]*Heart Research Center Göttingen University Medical Center Göttingen, Göttingen, Germany.*

[4]*Department of Cardiology & Pneumology, University Medical Center Göttingen, Göttingen, Germany.*

[5]*DZHK (German Center for Cardiovascular Research), partner site Göttingen, Germany.*

[6]*DZHK (German Center for Cardiovascular Research), partner site Berlin, Germany.*

[7]*Berlin Institute of Health (BIH), Berlin, Germany.*

[8]*Charité Universitätsmedizin, Berlin, Berlin, Germany.*

*To whom correspondence should be addressed: gotthardt@mdc-berlin.de.

Supplementary Figures:

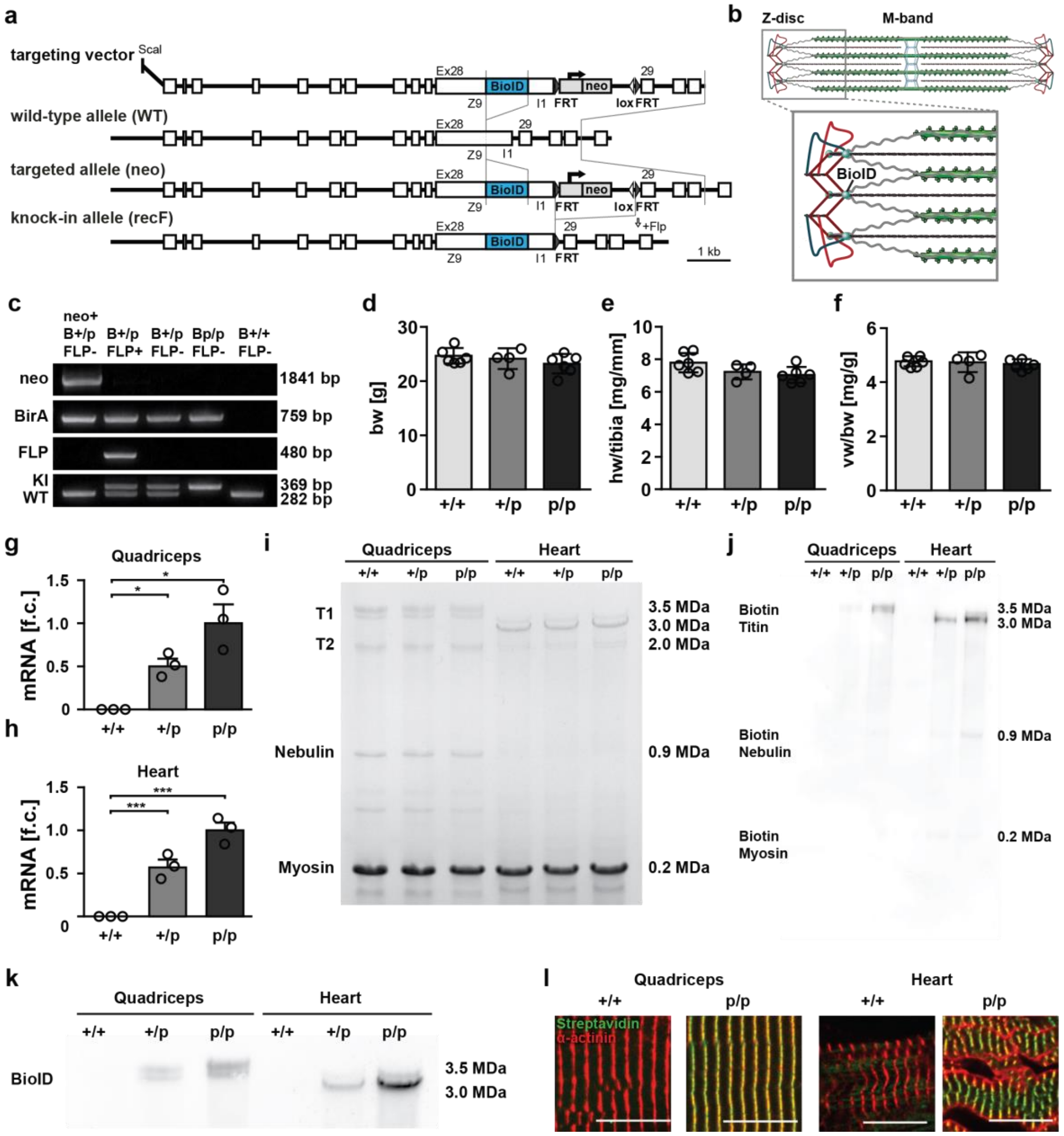

Supplementary Figure 1 | Cloning strategy and phenotypic analysis of titin-BioID knock-in mice. a, Targeting strategy for the insertion of BioID into titin's exon 28. **b**, Localization of BioID flanking the Z-disc (green myosin filament, grey titin). **c,** Genotyping of the TiZ-BioID knock-in mice with removal of the neo expression cassette and FLP transgene to produce the clean knock-in allele. **d-f,** Normal growth and cardiac dimensions in TiZ-BioID wildtypes, heterozygotes, and homozygotes (+/+, +/p, p/p). Left ventricle to bodyweight ratio (**d**), heart weight to tibia length ratio (**e**),

and ventricle weight to bodyweight (**f**) were unchanged between genotypes. Mean ± SD, n = 9. One-Way ANOVA, not significant. **g-j**, BioID mRNA and protein levels were intermediate in the heterozygotes with no background in wildtype quadriceps or cardiac muscle. Quadriceps BioID mRNA (**g**) and heart BioID mRNA levels (**h**) were below detection levels in wildtype and intermediate in heterozygous mice. (**g, h**) Mean ± SEM, n = 9. One-Way ANOVA $p < 0.0001$, Bonferroni post-test * $p<0.05$; *** $p<0.001$. **i**, Titin isoform expression was unchanged between genotypes as determined by agarose gel electrophoresis. **j**, The majority of biotinylated proteins was titin, with reduced biotin signal at the size of nebulin and myosin heavy chain. **k,** BirA titin was increased in homozygotes versus heterozygotes full length isoforms. **l**, Confocal images of cryosections from quadriceps and heart muscle of homozygous and wildtype mice stained for BirA (BioID). In homozygotes, the Z-disc α-actinin was decorated with biotinylated protein (green). Scale bar 10 µm. Source data are provided as a Source Data file.

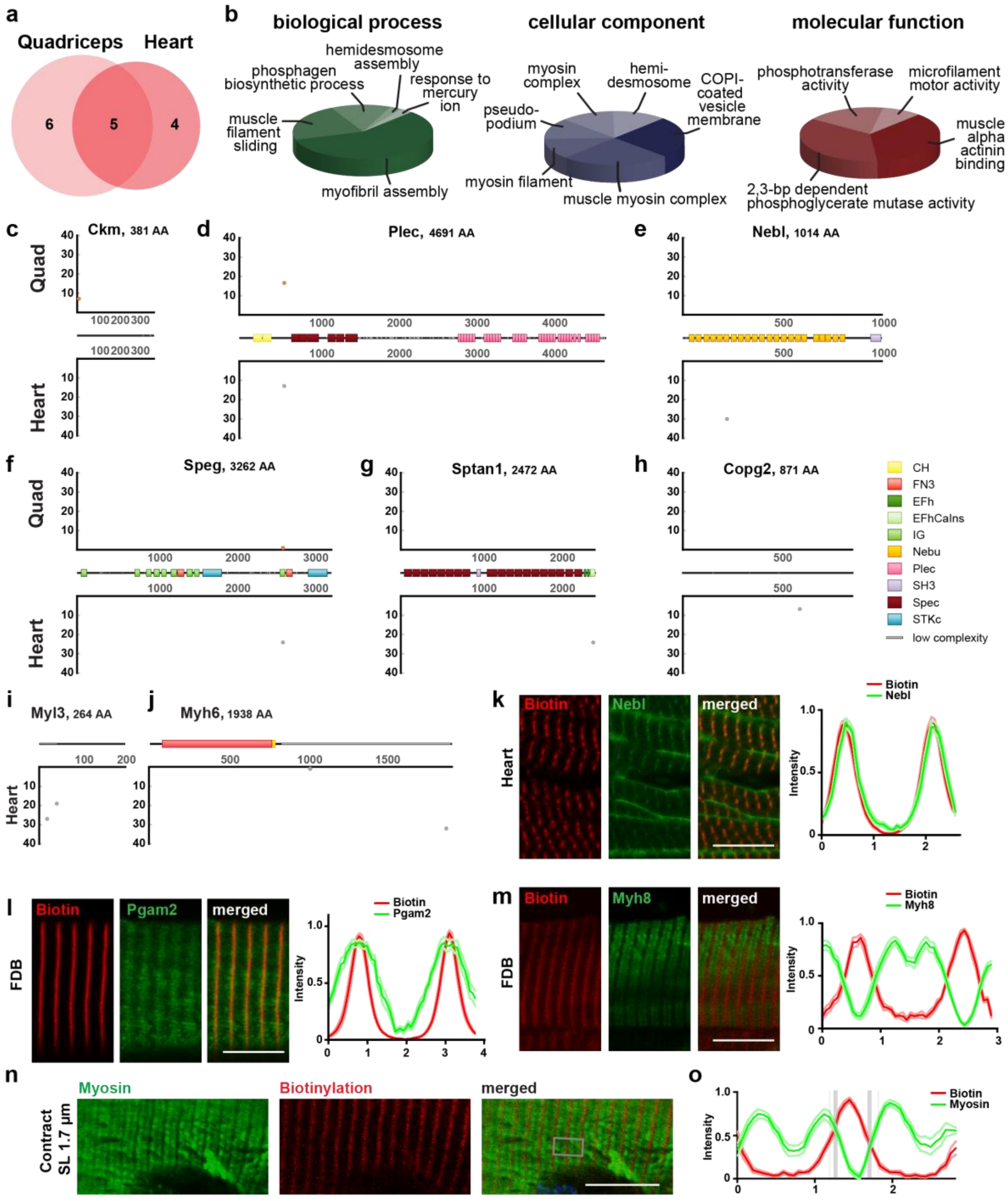

Supplementary Figure 2 | The titin-z-disc protein neighbourhood differs between heart and skeletal muscle and suggests specific interactions at the Z-I-transition. **a,** 15 proteins were biotinylated in striated muscle, of which 5 overlap between heart and skeletal muscle. **b**, Gene ontology analysis relates these proteins to myofibril assembly and muscle function, the hemidesmosome, metabolism and the Z-disc (alpha actinin binding). **c-j**, In the majority of biotinylated proteins exactly one site was

targeted with between 10 and 40 biotinylated peptides identified. For proteins biotinylated in both heart and quadriceps (**c, d**), site locations aligned. There was no preference for a specific domain. **k-m**, Immunofluorescence staining for proteins biotinylated at the Z-disc. **k**, In the heart, nebulette (Nebl) localized at the Z-disc, where it partially overlaped with biotinylated proteins enriched at the Z-disc. **l, m**) In flexor digitorum brevis muscle (FDB), Pgam2 and myosin heavy chain Myh8 partially localized at the Z-disc, but the majority of the protein localized at I-band and A-band for Pgam2 and Myh8, respectively. **n,** Co-staining of contracted FDB for myosin and biotinylated proteins. At low physiological sarcomere length of 1.7 µm, the myosin edge at the A/I junction did intersect with the Z-disc (overlap of red and green trace in **o**). Traces represent mean +/- SEM, n = 9. Scale bars 10 µm. Source data are provided as a Source Data file.

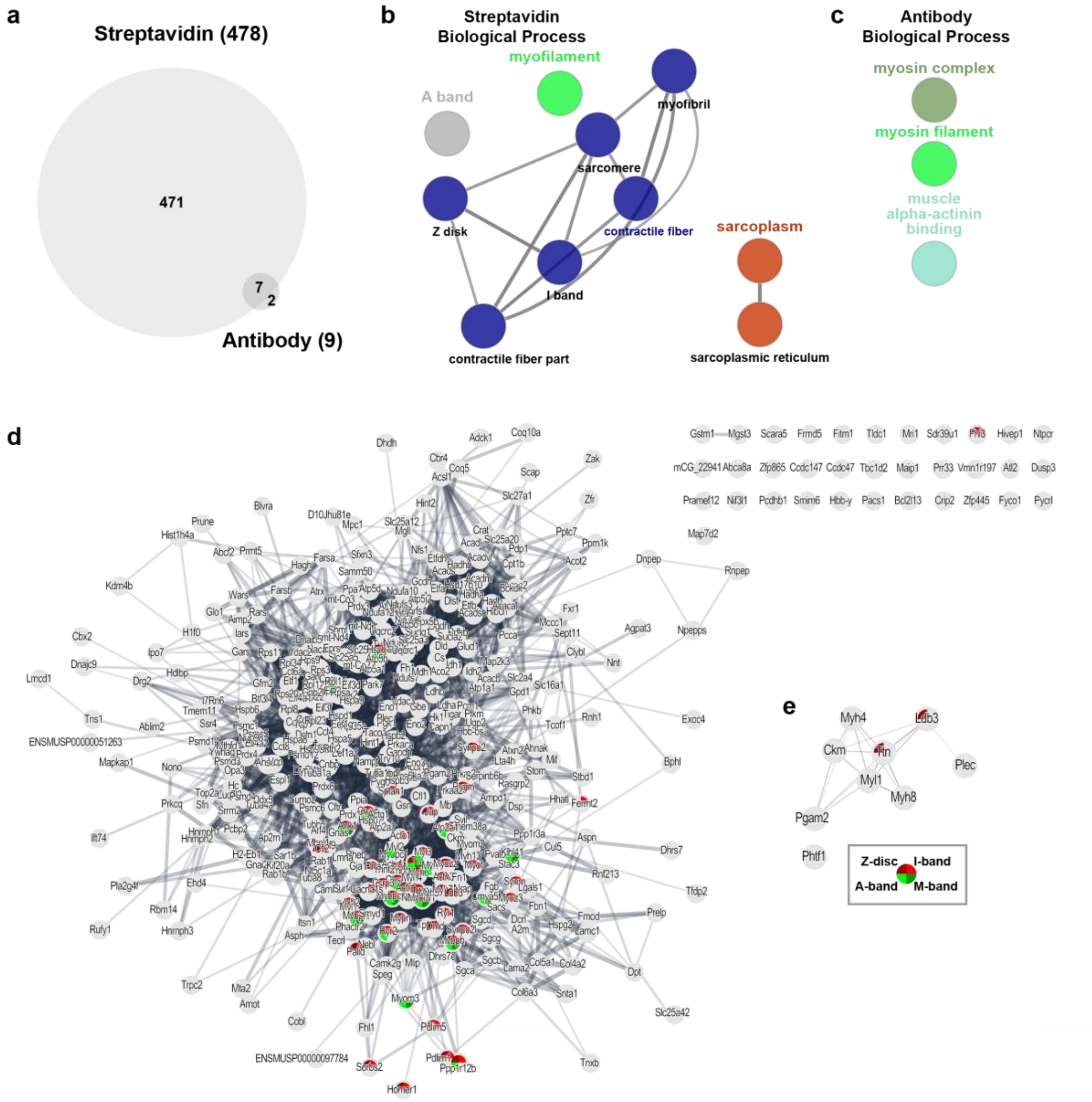

Supplementary Figure 3 | Extending the Z-disc to the sarcomere proteome using antibody versus streptavidin pulldowns. a, IP with streptavidin extends the sarcomere proteome to >450 proteins. These relate to all subregions of the myofibril (**b**) and the sarcoplasm. The antibody based identification of biotinylated peptides specifically identified myosins and α-actinin binding proteins (**c**). Interactome of cardiac proteins identified by streptavidin pulldown (**e**) vs. biotinylated proteins, of which only Phtf1 has not been linked to the Z-disc (**d**). Proteins associated with Z-disc and I-band are labelled in red. A- and M-band proteins in green. Source data are provided as a Source Data file.

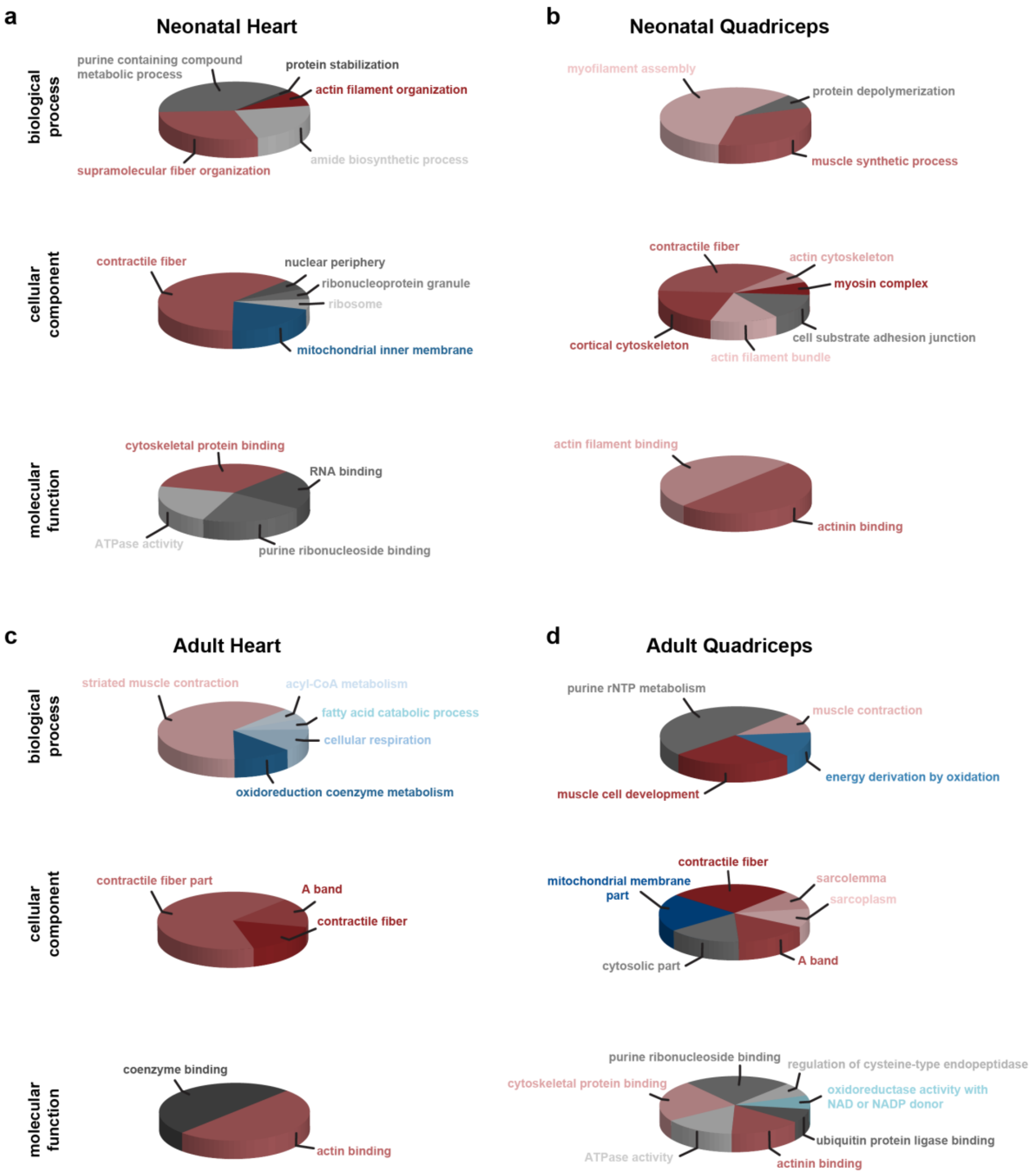

Supplementary Figure 4 | Gene ontology based comparison of neonatal and adult striated muscle myofilament proteomes. Sarcomere associated proteomes of neonatal versus adult heart and quadriceps relate to sarcomere biology (red, **a-d**). Adult tissue expressed sarcomeric proteins that link to energy metabolism (blue; **c, d**).

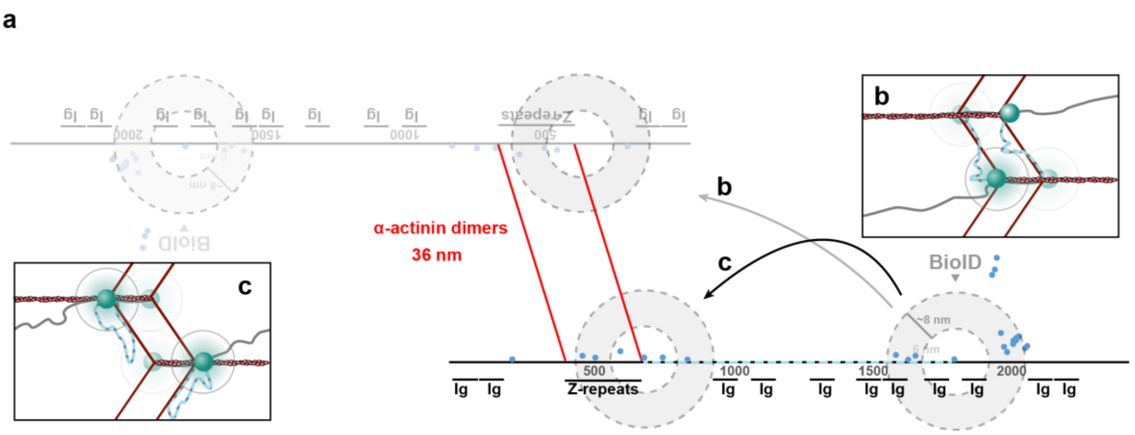

Supplementary Figure 5 | The biotinylation profile of Z-disc titin positions Ig8/9 at the Z-disc. a, Spacing of the biotinylation hotspot at titin Ig 8/9, the BioID insertion site and biotinylation profile (blue dots - compare Figure 1e). Amino acid positions (numbers) and positions of the immunoglobulin domains (black bars) are indicated. Actinin dimers (red) connect neighboring titin filaments (grey). Grey doughnuts indicate the range of biotinylation around the BioID. As the sarcomere contracts, the region emphasized with a dotted line in light blue is pushed towards the Z-disc core where α-actinin and titin interact. Here, titin could move along α-actinin towards the neighboring titin (**b,** light arrow) or form a hairpin to fold back to the proximal Z-repeat (**c,** dark arrow). The biotinylation sites proximal to the Z-repeats are only partially covered in **b** (upper doughnut) but fully covered in **c** (lower doughnut), consistent with back-looping of Ig8/9 to the proximal Z-repeat (**c**).

Supplementary Tables:

Supplementary Table 1: Sex and genotype distribution of offspring from heterozygous BioID matings (TiZ-BioID$^{p/+}$ X TiZ-BioID$^{p/+}$)

	**+/+**	**+/p**	**p/p**
Males	30	30	19
Females	16	39	25
**Total**	46	69	44

Supplementary Table 2: WikiPathway analysis of neonatal versus adult heart and quadriceps.

Neonatal Heart

GO Term	% Assoc Genes	Number Genes	Associated Genes Found
Striated Muscle Contraction	57.77778	26	[Actc1, Actg1, Actn2, Actn4, Des, Dmd, Mybpc3, Myh3, Myh4, Myh6, Myh7, Myl2, Myl3, Myl4, Myom1, Myom2, Neb, Smpx, Tcap, Tnni1, Tnni3, Tnnt2, Tpm1, Tpm3, Ttn, Vim]
TNF-alpha NF-kB Signaling Pathway	14.28571	27	[Csnk2b, Ddx3x, Eif4a3, Fbl, Flna, Gsk3b, Hsp90aa1, Hsp90ab1, Hspb1, Ktn1, Mcm7, Ppp1r13l, Prkaca, Psmb5, Psmc1, Psmc2, Psmc3, Psmd12, Psmd3, Rpl30, Rps11, Tab2, Ywhab, Ywhae, Ywhag, Ywhah, Ywhaz]
Electron Transport Chain	33.00971	34	[Atp5a1, Atp5b, Atp5c1, Atp5d, Atp5f1, Atp5h, Atp5j2, Atp5o, Cox4i1, Cox5a, Cox5b, Cox6c, Cox7b, Ndufa1, Ndufa10, Ndufa2, Ndufa5, Ndufa7, Ndufa9, Ndufb4, Ndufb9, Ndufs1, Ndufs3, Ndufs6, Ndufs8, Ndufv1, Ndufv2, Ndufv3, Sdha, Sdhb, Slc25a5, Uqcrc1, Uqcrc2, Uqcrq]
mRNA processing	14.34783	66	[Aimp1, Alyref2, Auh, Ddx3x, Ddx5, Dhx9, Dnajc8, Eftud2, Eif4a3, Elavl1, Fbl, Fmr1, Fxr1, G3bp1, Hnrnpa0, Hnrnpa1, Hnrnpa2b1, Hnrnpa3, Hnrnpc, Hnrnpd, Hnrnph1, Hnrnph2, Hnrnpk, Hnrnpl, Hnrnpm, Hnrnpr, Hnrnpu, Igf2bp1, Igf2bp3, Ilf3, Matr3, Mbnl1, Ncl, Nifk, Nono, Npm1, Pabpc1, Pabpc4, Pcbp1, Pcbp2, Prmt1, Prpf8, Ptbp1, Rpl11, Rpl12, Rpl22, Rpl9, Rps11, Rps14, Rps20, Rps27, Rps7, Sf3a2, Sfpq, Smc1a, Snrpd1, Snrpd2, Snrpf, Srrm1, Srsf10, Srsf6, Ssb, Syncrip, Tardbp, Tnrc6c, U2af2]
Focal Adhesion-PI3K-Akt-mTOR-signaling pathway	8.615385	28	[Col11a1, Col1a1, Col1a2, Col4a1, Col4a2, Col5a1, Col6a2, Eif4b, Elavl1, Fn1, Gnb2, Gsk3b, Hsp90aa1, Hsp90ab1, Hsp90b1, Jak1, Kras, Lama2, Lama4, Lamb1, Lamb2, Lamc1, Prkaa2, Rab10, Rab11b, Slc2a1, Tnxb, Vwf]
Focal Adhesion	17.83784	33	[Actb, Actg1, Actn1, Cav3, Col11a1, Col1a1, Col1a2, Col4a1, Col4a2, Col5a1, Col6a2, Crk, Crkl, Flna, Fn1, Gsk3b, Ilk, Lama2, Lama4, Lamb1, Lamb2, Lamc1, Myl6, Ppp1r12a, Rac1, Rap1b, Rhoa, Tln1, Tnxb, Vasp, Vcl, Vwf, Zyx]

Adult Heart

GO Term	% Assoc Genes	Number Genes	Associated Genes Found
Fatty Acid Beta Oxidation	38.23529	13	[Acadl, Acadm, Acads, Acadvl, Acat1, Acsl1, Cpt1b, Crat, Dld, Gcdh, Hadh, Hadha, Hadhb]
Glycolysis and Gluconeogenesis	25.4902	13	[Aldoa, Dld, Eno1, Eno3, Gapdh, Hk1, Ldha, Ldhb, Mdh1, Mpc1, Pcx, Pfkm, Pgk1]
Striated Muscle Contraction	37.77778	17	[Actc1, Actg1, Actn2, Mybpc3, Myh3, Myh4, Myh6, Myh7, Myl2, Myl3, Myom1, Myom2, Tcap, Tnni3, Tnnt2, Tpm1, Ttn]
Electron Transport Chain	12.62136	13	[Atp5a1, Atp5b, Atp5c1, Cox5b, Ndufa10, Ndufs1, Ndufs7, Ndufv1, Slc25a4, Slc25a5, Uqcrc1, Uqcrc2, Uqcrfs1]
TCA Cycle	38.70968	12	[Aco2, Cs, Dld, Dlst, Fh1, Idh2, Mdh1, Ogdh, Pcx, Pdp1, Sucla2, Suclg1]
Amino Acid metabolism	17.70833	17	[Acadm, Aco2, Acss1, Cs, Dld, Dlst, Farsb, Fh1, Glud1, Hadh, Hibch, Ldha, Mccc1, Mdh1, Ogdh, Pcx, Suclg1]

Neonatal Quadriceps

GO Term	% Assoc Genes	Number Genes	Associated Genes Found
Cytoplasmic Ribosomal Proteins	13.59223	14	[Rpl11, Rpl12, Rpl22, Rplp0, Rps12, Rps14, Rps17, Rps2, Rps20, Rps27, Rps27a, Rps3, Rps7, Rpsa]
Striated Muscle Contraction	46.66667	21	[Acta1, Actc1, Actg1, Actn2, Actn4, Des, Dmd, Mybpc1, Myh3, Myh4, Myh6, Myh7, Myh8, Myom1, Myom2, Smpx, Tcap, Tnni1, Tnni2, Tpm1, Ttn]
TNF-alpha NF-kB signaling Pathway	5.820106	11	[Fbl, Flna, Hsp90ab1, Hspb1, Lrpprc, Prkaca, Psmc1, Psmc3, Rack1, Tab2, Ywhae]
mRNA processing	6.73913	31	[Ddx39b, Dhx9, Eif3g, Elavl1, Fbl, Fxr1, Hnrnpa2b1, Hnrnpc, Hnrnph2, Hnrnpk, Hnrnpl, Ncl, Nifk, Nol8, Nono, Pabpc1, Pabpc4, Pcolce, Prmt1, Ptbp1, Rpl11, Rpl12, Rpl22, Rps14, Rps20, Rps27, Rps7, Sfpq, Slc25a4, Srsf6, Tardbp]
MAPK signaling pathway	6.918239	11	[Crk, Crkl, Flna, Hspa1a, Hspa5, Hspa8, Hspb1, Hspb2, Prkaca, Rap1b, Tab2]
Regulation of Actin Cytoskeleton	10.52632	16	[Actb, Actg1, Cfl1, Cfl2, Crk, Fn1, Gsn, Msn, Myh10, Pip4k2c, Ppp1r12a, Rdx, Rhoa, Rock2, Rras2, Vcl]
Focal Adhesion	11.35135	21	[Actb, Actg1, Col6a2, Crk, Crkl, Flna, Fn1, Ilk, Lama2, Lama4, Lamb1, Lamb2, Myl6, Ppp1r12a, Rap1b, Rhoa, Rock2, Tln1, Tnxb, Vcl, Zyx]

Adult Quadriceps

GO Term	% Assoc Genes	Number Genes	Associated Genes Found
Glycolysis and Gluconeogenesis	21.56863	11	[Aldoa, Eno1, Eno2, Gapdh, Ldha, Pcx, Pdhb, Pfkm, Pgam2, Pgk1, Slc2a4]
Cytoplasmic Ribosomal Proteins	14.56311	15	[Rpl11, Rpl12, Rpl15, Rpl22, Rpl34, Rpl6, Rpl8, Rps11, Rps2, Rps20, Rps27a, Rps3, Rps6ka3, Rps7, Rpsa]
Striated Muscle Contraction	40	18	[Acta1, Actc1, Actn2, Actn3, Des, Dmd, Mybpc1, Mybpc2, Myh1, Myh3, Myh4, Myl1, Myom1, Myom2, Neb, Tcap, Tpm1, Ttn]
TNF-alpha NF-kB Signaling Pathway	6.878307	13	[Ddx3x, Flna, Hsp90ab1, Hspb1, Kpna6, Prkaca, Psmc1, Psmd12, Psmd3, Rack1, Rpl6, Rpl8, Rps11]
Oxidative phosphorylation	19.35484	12	[Atp5a1, Atp5b, Atp5d, Atp5f1, Atp5h, Atp5j2, Ndufa10, Ndufa6, Ndufa9, Ndufs3, Ndufs7, Ndufv1]
Electron Transport Chain	16.50485	17	[Atp5a1, Atp5b, Atp5c1, Atp5d, Atp5f1, Atp5h, Atp5j2, Ndufa10, Ndufa12, Ndufa6, Ndufa9, Ndufs3, Ndufs7, Ndufv1, Slc25a4, Uqcrc1, Uqcrfs1]
