# Supplementary material for: Deconstructing sarcomeric structure–function relations in titin-BioID knock-in mice
Source: Nat Commun. 2020 Jun 19;11:3133. doi: 10.1038/s41467-020-16929-8 (PMC7305127; doi:10.1038/s41467-020-16929-8)
Supplement: Supplementary file 3 — Reporting Summary [file 41467_2020_16929_MOESM3_ESM.pdf]

nature research

Corresponding author(s): Michael Gotthardt

Last updated by author(s): May 19, 2020

Reporting Summary

Nature Research wishes to improve the reproducibility of the work that we publish. This form provides structure for consistency and transparency in reporting. For further information on Nature Research policies, see our Editorial Policies and the Editorial Policy Checklist.

Statistics

For all statistical analyses, confirm that the following items are present in the figure legend, table legend, main text, or Methods section.

n/a	Confirmed	
☐	☒	The exact sample size (n) for each experimental group/condition, given as a discrete number and unit of measurement
☐	☒	A statement on whether measurements were taken from distinct samples or whether the same sample was measured repeatedly
☐	☒	The statistical test(s) used AND whether they are one- or two-sided<br>*Only common tests should be described solely by name; describe more complex techniques in the Methods section.*
☒	☐	A description of all covariates tested
☒	☐	A description of any assumptions or corrections, such as tests of normality and adjustment for multiple comparisons
☒	☐	A full description of the statistical parameters including central tendency (e.g. means) or other basic estimates (e.g. regression coefficient) AND variation (e.g. standard deviation) or associated estimates of uncertainty (e.g. confidence intervals)
☐	☒	For null hypothesis testing, the test statistic (e.g. F, t, r) with confidence intervals, effect sizes, degrees of freedom and P value noted<br>*Give P values as exact values whenever suitable.*
☒	☐	For Bayesian analysis, information on the choice of priors and Markov chain Monte Carlo settings
☒	☐	For hierarchical and complex designs, identification of the appropriate level for tests and full reporting of outcomes
☒	☐	Estimates of effect sizes (e.g. Cohen's d, Pearson's r), indicating how they were calculated

Our web collection on statistics for biologists contains articles on many of the points above.

Software and code

Policy information about availability of computer code

Data collection	Xcalibur version 3.0.63, Xcalibur version 4.0.27.10, LAS X 33.5.5
Data analysis	GraphPad Prism6, MaxQuant software package (v1.5.1.2), Perseus software (v1.6.2.1), Fuji Software (ImageJ2)

For manuscripts utilizing custom algorithms or software that are central to the research but not yet described in published literature, software must be made available to editors and reviewers. We strongly encourage code deposition in a community repository (e.g. GitHub). See the Nature Research guidelines for submitting code & software for further information.

Data

Policy information about availability of data

All manuscripts must include a data availability statement. This statement should provide the following information, where applicable:

- Accession codes, unique identifiers, or web links for publicly available datasets
- A list of figures that have associated raw data
- A description of any restrictions on data availability

The data that support the findings of this study are available from the article and Supplementary Information files, or from the corresponding author upon request. Proteomics data have been deposited to PRIDE server under accession code PXD017341. Uniprot database MOUSE.2017-01; STRING Database Version 10.5; Gene Ontology Database 10.5281/zenodo.2529950. The source data underlying Figures 1b,e, 2a–g,i,k, 3a-c, 5a-i and Supplementary Figures 1d-k, 2a-j, 3a, and 5a-i are provided as a Source Data file.

Field-specific reporting

Please select the one below that is the best fit for your research. If you are not sure, read the appropriate sections before making your selection.

- [x] Life sciences
- [ ] Behavioural & social sciences
- [ ] Ecological, evolutionary & environmental sciences

For a reference copy of the document with all sections, see nature.com/documents/nr-reporting-summary-flat.pdf

Life sciences study design

All studies must disclose on these points even when the disclosure is negative.

Sample size	N=3-6 for expression analysis and 3-10 for functional analysis based on examples in the literature. doi: 10.1038/nm.2693
Data exclusions	None
Replication	Experiments were replicated once or twice with technical replicates for the MS analysis and biological replicates for stainings, Replicates were successful.
Randomization	Animals were allocated to experimental groups by genotype. As there were no treatment groups, there was no additional randomization.
Blinding	For expression analysis, blinding was not possible to enable orderly loading of the gels. For physiology measurements, investigators were blinded. For immunofluorescence analysis staining revealed genotypes, so that blinding was not possible.

Reporting for specific materials, systems and methods

We require information from authors about some types of materials, experimental systems and methods used in many studies. Here, indicate whether each material, system or method listed is relevant to your study. If you are not sure if a list item applies to your research, read the appropriate section before selecting a response.

Materials & experimental systems

n/a	Involved in the study	
[ ]	[x]	Antibodies
[x]	[ ]	Eukaryotic cell lines
[x]	[ ]	Palaeontology and archaeology
[ ]	[x]	Animals and other organisms
[x]	[ ]	Human research participants
[x]	[ ]	Clinical data
[x]	[ ]	Dual use research of concern

Methods

n/a	Involved in the study	
[x]	[ ]	ChIP-seq
[x]	[ ]	Flow cytometry
[x]	[ ]	MRI-based neuroimaging

Antibodies

Antibodies used

1:5000 BirA antibody from BioFront Technologies (Chicken Polyclonal Ab to E. coli Biotin Ligase/BirA; order number: BID-CP-100, clone R118G)
1:1000 Streptavidin-HRP conjugate from GE Healthcare, order number: RPN1231V
1:50 anti-α-actinin, Sigma-Aldrich, clone EA-53; order number A7811
1:50 anti-Myh8, Invitrogen, polyclonal, order number: PA5-72846
1:50 anti-Myosin; Sigma-Aldrich, clone: MY-32, order number: M4276
1:50 anti-Nebl, SCBT, clone: G-9, order number: sc-393784
1:50 anti-Pgam2, Abcam, polyclonal, order number: ab97800
1:200 goat anti-mouse STAR580 from Abberior (order number: 2-0002-005-1)
1:200 goat anti-rabbit STAR580 from Abberior (order number: 2-0012-005-8)
1:200 Streptavidin STAR635P from Abberior (order number: 2-0205-007-0)
1:5000 ECL Rabbit IgG, HRP-linked whole Ab (from donkey) Amersham (order number: NA934V)

Validation

BirA antibody were purchased from BioFront Technologies (Chicken Polyclonal Ab to E. coli Biotin Ligase/BirA; order number: BID-CP-100, clone R118G), Manufacturers validation: “Working dilution Immunofluorescence 1:5000”
Streptavidin-HRP conjugate from GE Healthcare, order number: RPN1231V, Manufacturers validation: “The control system used was the detection of monoclonal anti-tubulin. We have found in our laboratories that dilutions of 1:2000 for monoclonal anti-tubulin; 1:2500 for anti-mouse Ig, biotinylated; and 1:5000 for Streptavidin biotinylated HRP complex are suitable for the detection of 5 ng of tubulin on Hybond ECL membrane, exposed to Hyperfilm™ ECL for 5 minutes”
anti-α-actinin, Sigma-Aldrich, clone EA-53; order number A7811, Manufacturers validation: “Immunohistochemistry: a minimum titer of 1:800 was determined by indirect immunoperoxidase labeling of protease-digested, formalin-fixed, paraffin-embedded sections of

	human skeletal or cardiac muscle."<br>anti-Myh8, Invitrogen, polyclonal, order number: PA5-72846; Manufacturers validation: tested dilution of 20 ug/ml in Immunofluorescence ... 1 mg/ml stock concentration"<br>anti-Myosin; Sigma-Aldrich, clone: MY-32, order number: M4276; Manufacturers validation: "indirect immunofluorescence: 1:20 using human or animal sletetal muscle"<br>anti-Nebl, SCBT, clone: G-9, order number: sc-393784; Manufacturers validation: "Nebulette (G-9) is recommended for detection of Nebulette of mouse, rat and human origin by ... immunofluorescence (starting dilution 1:50, dilution range 1:50-1:500)"<br>anti-Pgam2, Abcam, polyclonal, order number: ab97800; Manufacturers validation: "Our Abpromise guarantee covers the use of ab97800 in the following tested applications. The application notes include recommended starting dilutions; optimal dilutions/ concentrations should be determined by the end user. ICC/IF: 1:100-1:200"

Animals and other organisms

Policy information about studies involving animals; ARRIVE guidelines recommended for reporting animal research

Laboratory animals	Mice, male, 129/SV, 100+ days old or newborn, Animals were housed in individually ventilated cages with free access to food and water, constant temperature 22±2°C and 55±10% humidity, and a 12h:12h light/dark cycle.
Wild animals	No wild animals were used.
Field-collected samples	No field collected samples were used.
Ethics oversight	All experiments involving animals were performed following the rules for Animal Welfare of the German Society for Laboratory Animal Science and received ethical approvaled by the Landesamt für Gesundheit und Soziales (LAGeSo, Berlin).

Note that full information on the approval of the study protocol must also be provided in the manuscript.
